# Supplementary material for: The Mycobacterium smegmatis HesB Protein, MSMEG_4272, Is Required for In Vitro Growth and Iron Homeostasis
Source: Microorganisms. 2023 Jun 14;11(6):1573. doi: 10.3390/microorganisms11061573 (PMC10301553; doi:10.3390/microorganisms11061573)
Supplement: Supplementary file 1 [file microorganisms-11-01573-s001.zip › microorganisms-2429716-supplementary.pdf]

microorganisms MDPI

Supplementary Materials:

Table S1: Strains and plasmids used in this study.

Strain Names	Description	Source or reference
mc²155	*M. smegmatis* strain (ATCC: 700084)	SB Snapper PubMed: 2082148
*4272*ID	*M. smegmatis* mc²155 mutant in which chromosomally encoded MSMEG_4272 is fused to a C-terminal ID-tagged	This study
*4272*ID::HIV2Pr	Derivative of the *4272*ID strain containing the pMC1s::HIV2Pr vector; Km^r	This study
WT::empty	Derivative of *M. smegmatis* mc²155 strain with the pLJR962 plasmid integrated at the attB site; Km^r	This study
WT::sgRNA	Derivative of *M. smegmatis* mc²155 strain with the pSG4272 plasmid integrated at the attB site; Km^r	This study
*4272*ID::empty	Derivative of the *4272*ID strain with the pLJR962 plasmid integrated at the attB site; Km^r	This study
*4272*ID::sgRNA	Derivative of the *4272*ID strain with the pSG4272 plasmid integrated at the attB site; Km^r	This study
**Plasmid Names**	**Description**	**Source or reference**
p2NIL	Cloning vector, oriE, Kan^R	Addgene plasmid 20188
pGOAL17	pBR322 derivative carrying marker genes (P_{Ag85}-lacZ; P_{hsp60}-sacB) flanked by two PacI sites. oriE, Amp^R	Addgene plasmid 20189
p2NIL4272LS	Suicide delivery vector containing upstream and downstream homologous regions designed to introduce a 246 bp deletion into MSMEG_4272 and PacI cassette from pGOAL17; Km^R	This study
pdacB_SsrA-tag	Plasmid containing an ID-tag consisting of a myc-tag, a TB SsrA-tag, a HIV-2 protease cutting site, and a flag-tag flanked on the 5′- and 3′-ends by HindIII and SacI restriction sites, Kan^R	Wei et al., 2011 [38]
pJET_4272ssr	pJET 2.1 Blunt derivative containing *MSMEG_4272* and 300 bp upstream, accompanied by an ID-tag, Amp^R	This study
pJET_4272ID	pJET_4272ssr derivative, in which 881 bp of the *MSMEG_4272* downstream region is fused to the C-terminal region of the ID-tag, Amp^R	This study
pJET_4272_up	pJET 2.1 Blunt derivative containing 1500 bp upstream of *MSMEG_4272*, Amp^R	L. Ngakane
pN_4272ID	p2NIL derivative, containing an ID-tagged *MSMEG_4272*, including 1500 bp upstream and 881 bp of the downstream region, Kan^R	This study
pNG4272ID	Suicide delivery vector, pN_4272ID derivative containing lacZ and SacB selectable markers cloned into the PacI site, Kan^R	This study
pMC1s::WT-HIV2Pr	Integrating mycobacterial vector, HIV-protease under the control of a tetracycline-responsive promoter, Km^r	Wei et al., 2011 [38]

pLJR962	Integrating mycobacterial vector, Sth1 dCas9 under the control of a tetracycline-responsive promoter, Km^r, L5 Int attP for *M. smegmatis*	[47], Addgene plasmid 115162
pSG4272	PLJR962 derivative containing the MS4272_CRISPRi directed sgRNA in the BsmBI restriction enzyme site. Km^r	T. de Wet

Table S2: Primers used in this study.

**Primer name**	**Sequence** (5′ – 3′) *	**Binding site**	**Product**
S4272UF	CAGGATCCGTCATGTCGGCTCCTTCAC	1481 bp upstream of the start of *MSMSEG_4272*	1500 bp upstream homologous region used for *MSMEG_4272* knock-out construct (p2NIL4272LS)
S4272UR	CGAATTCGCGTCCTGAACAGTCAT	19 bp downstream of the start of *MSMSEG_4272*	
S4272DF	CGAATTCGATCGACTTCGTCGACACCAT	93 bp from the 3′-end of *MSMEG_4272*	1578 bp downstream homologous region used for *MSMEG_4272* knock-out construct (p2NIL4272LS)
S4272DR	GCAAGCTTATCCCACTCGTAGTCGTTGG	1485 bp downstream of the 3′-end of *MSMEG_4272*	
Fw_4272_up	CCATGCGTCCACGGCGGAATCCG-CAGCG	300 bp from the start of *MSMEG_4272*	*MSMEG_4272* amplification, including 300 bp upstream of *MSMEG_4272*, and removal of the *MSMEG_4272* native stop codon; 15 bp overlap with ID-tag N-terminal region.
Rev_4272_ID	GATCAGCTTCTGCTCGTTGAA-GCTGTCGCC	3 bp from 3′-end of *MSMEG_4272*.	
Fw_IDtag	GAGCAGAAGCTGATCTCGGAG	5′ – end of ID-tag in dacB_SsrA-tag.	Amplifies ID-tag and introduces a C-terminal HindIII restriction site.
Rev_IDtag	AAGCTTCTTGTCGTCGTCGTCCTT-GTAGTC	3′ – end of ID-tag in dacB_SsrA-tag	
F_ID_4272DS	ACGACGACGACAAGTGACGGCCGG-TACTGCTT	24 bp downstream of *MSMEG_4272* coding region.	860 bp downstream homologous region used for inserting ID-tag downstream of *MSMEG_4272* (pNG4272ID). Fragment used as a probe during Southern blotting procedure.
R4272_881DS	GCGGCCGCGTGGCACCCTTCATTTGTTC	881 bp downstream of MSMEG_4272, introducing a NotI restriction site.	
4272delF1	TCTTGTCGTCGTCGTT-GTTC	266 bp upstream of the start of *MSMEG_4272*	Primers used to screen for *MSMEG_4272* deletion.<br>Wild type: 427 bp<br>Mutant: 791 bp
4272delR1	AACAGGTTGTAGCGCAGACC	161 downstream of the start of *MSMEG_4272*	
4272delR2	ACGCGATCCAGGAGTATCTG	414 bp downstream of the 3′-end of *MSMEG_4272*	
FMsmeg4272	TCTTGTCGTCGTCGTTGTTC	226 bp upstream of *MSMEG_4272* gene sequence.	Primers used to screen for incorporation of ID-tag on the C-terminus of *MSMEG_4272*.<br>Wild type: 776 bp<br>Mutant: 857 bp
RMsmeg4272	ACGCGATCCAGGAGTATCTG	414 bp downstream of *MSMEG_4272* gene sequence.	
rtsmSiga-r1	GTATCCCGGTGCATGGTC	1035 bp downstream of the start of *M. smegmatis* SigA	sigA-specific primer used for cDNA synthesis

RT_4272	GTTGAACTGTCGCCGCAGG	334 bp downstream of the start of *MSMEG_4272*	MSMEG_4272-specific primer used for cDNA synthesis
RT4272-F2	CGGTCTGCGCTACAACCTG	144 bp downstream from the start of *MSMEG_4272*	qPCR primer: 178 bp product for quantitation of MSMEG_4272 transcript levels
RT4272-R2	GGCGTTGGGGTTGTCGATC	321 bp downstream of the start of *MSMEG_4272*	
RTSIGA_F2	ACCGCGCCAAAAACCATCTG	680 bp downstream of the start of *M. smegmatis* SigA	qPCR primer: 80 bp product for quantitation of sigA transcript levels
RTSIGA_R2	TGGATGAGGTCGAGGAACGC	760 bp downstream of the start of *M. smegmatis* SigA	

*Restriction enzyme sites used for cloning are underlined

Table S3: PCR screening results following two-step selection to generate Δ*MSMEG_4272*

Experiment	Colonies Screened	Wild-Type (WT)	SCOs	Mutant
1	27	16	11	0
2	50	12	38	0

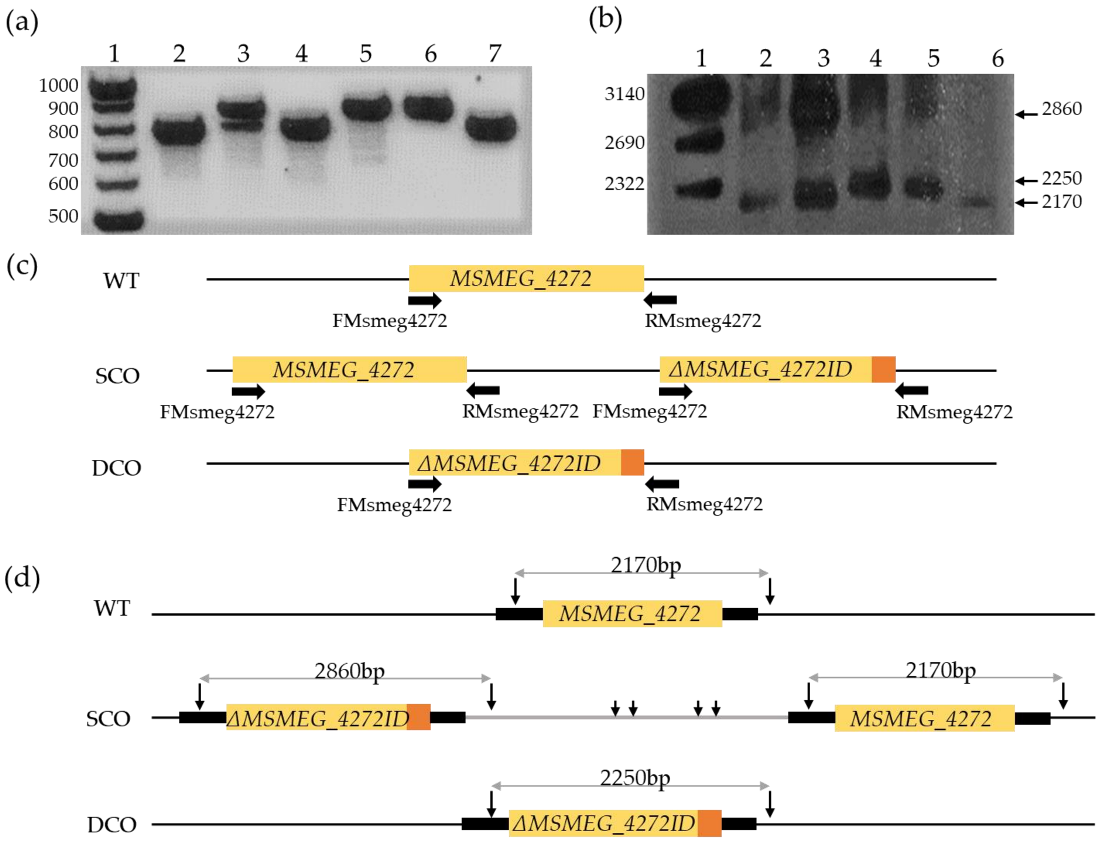

Figure S1: Confirmation of the *M. smegmatis* ΔMSMEG_4272ID mutant strain genotypes. (**a**) Agarose gel electrophoresis of the colony PCR products for *M. smegmatis* mc^{2}155 (lane 2), *4272*ID SCO (lane 3) and the potential *4272*ID DCO mutants (lane 4 –7). (**b**) Southern blot analysis: *M. smegmatis* mc^{2}155 (lane 2 & 6), *4272*ID SCO (lane 3), and two *4272*ID DCO's (lane 4 & 5). (**c**) PCR screening approach to differentiate between the ΔMSMEG_4272ID mutant allele (857bp) and the MSMEG_4272 wild-type reverted allele (776bp). Arrows indicate the binding region of primers (binding of specific primers are indicated) (**d**) Graphical presentation of the strategy for Southern blot genotype for *M. smegmatis*

mc[2]155 (WT), *M. smegmatis* SCO, and *M. smegmatis* DCO mutants. Arrows indicate the cut sites of the restriction enzyme site, PvuII. The MSMEG_4272 downstream region, amplified with F_ID_4272DS and R4272_881DS, was used as a probe during southern blotting. The gene sizes are not illustration to scale.

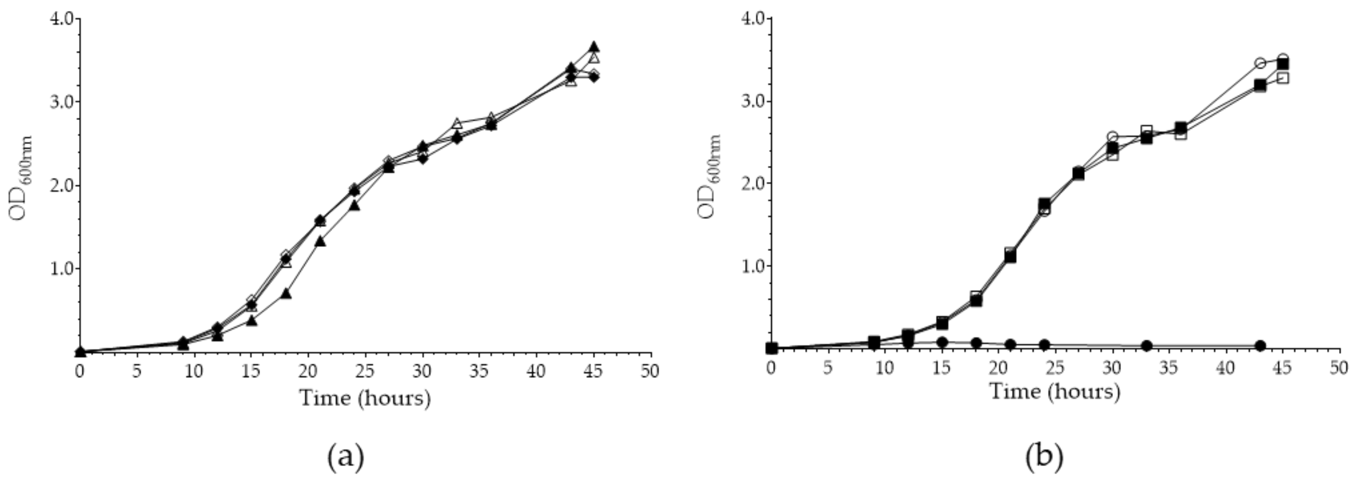

Figure S1: Optimization of growth for CRISPRi mediated *MSMEG_4272* gene silencing in *M. smegmatis* strains under standard culture conditions. (**a**) Growth of WT::empty, with and without Atc (solid & open diamond, respectively), and WT::sgRNA, with and without Atc (solid & open triangle, respectively). (**b**) Growth of *4272*ID::empty, with and without Atc (solid & open square, respectively), and *4272*ID::sgRNA, with and without Atc (solid & open circle, respectively). Data (A & B) represents one biological replicate.

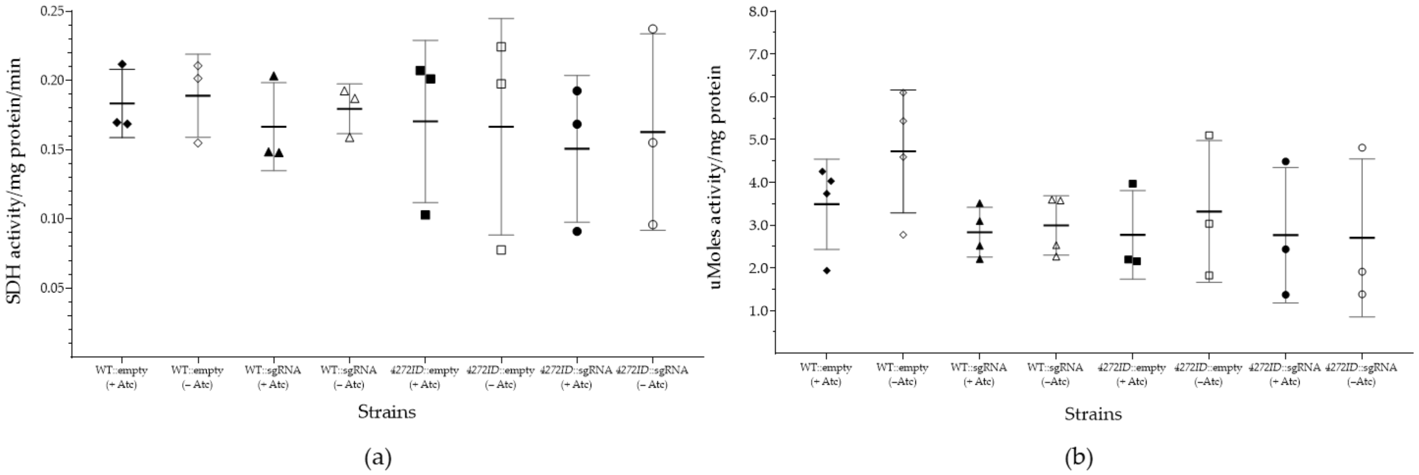

Figure S2: Activity of Fe-S cluster containing enzymes in *M. smegmatis* strains. The *M. smegmatis* strains WT::empty, WT::sgRNA, *4272*ID::empty, and *4272*ID::sgRNA were cultured in the presence and absence of Atc. Aliquots were taken after the cultures reached an OD_{600} of ~0.6 –0.8. The activity of (**a**) succinate dehydrogenase and (**b**) aconitase was measured per minute, for each milligram protein. Data represents the average, and error bars represent standard deviation, of three biological replicates. GraphPad Prism 8 software was used to determine the p-values using an unpaired t-test. Where appropriate, statistical significance is indicated by $p<0.05$ (*), $p<0.01$ (**) and $p<0.001$ (***).

Table S4: Minimum inhibitory concentration for CRISPRi mediated *MSMEG_4272* gene silencing in various *M. smegmatis* strains. Clofazimine and DMNQ MIC ranges are displayed in accordance with the standardised Broth-microdilution methods. The MIC ranges for Isoniazid are displayed as an extended range.

	**Minimum Inhibitory Concentration ranges**		
***M. smegmatis* strains**	**Clofazimine (µg/mL)**	**DMNQ (µg/mL)**	**Isoniazid (µg/mL)**
WT::empty (+Atc)	0.5 - 1	55.86 – 111.12	32 - 256
WT::empty (–Atc)	0.5 - 1	55.86 – 111.12	32 - 256
WT::sgRNA (+Atc)	0.25 – 0.5	27.93 - 55.86	8 - 32
WT::sgRNA (–Atc)	0.5 - 1	55.86 – 111.12	32 - 256
*4272*ID::empty (+Atc)	0.5 - 1	13.96 - 27.93	16 - 128
*4272*ID::empty (–Atc)	0.5 - 1	13.96 - 27.93	16 - 128
*4272*ID::sgRNA (+Atc)	0.25 - 0.5	13.96 - 27.93	1 - 16
*4272*ID::sgRNA (–Atc)	1 - 2	55.86 – 111.12	8 - 256

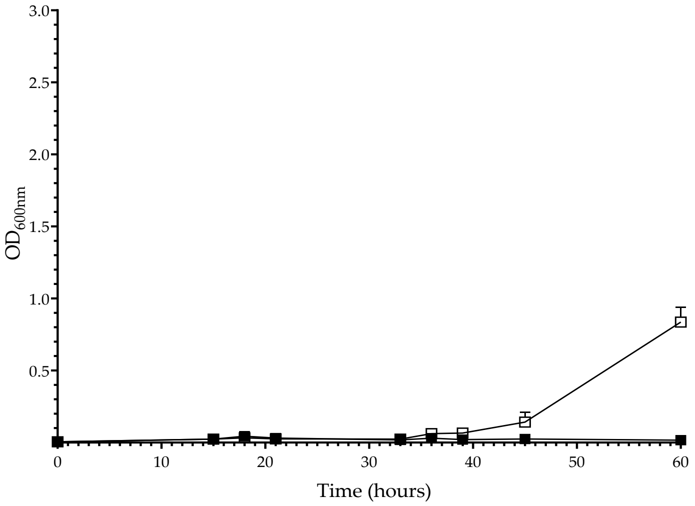

Figure S3: Growth of CRISPRi mediated MSMEG_4272 gene silencing in *M. smegmatis* strains under iron limiting culture conditions in modified MM Media. Growth of *4272*ID::sgRNA in modified MM media supplemented with 2 µM iron, with and without Atc (solid & open square, respectively). Data represent the mean of three biological replicates and error bars indicate standard deviation.
